# PEG-mediated osmotic stress induces premature differentiation of the root apical meristem and outgrowth of lateral roots in wheat

**DOI:** 10.1093/jxb/eru255

**Published:** 2014-06-16

**Authors:** Hongtao Ji, Ling Liu, Kexue Li, Qingen Xie, Zhijuan Wang, Xuhua Zhao, Xia Li

**Affiliations:** The State Key Laboratory of Plant Cell & Chromosome Engineering, Center for Agricultural Research Resources, Institute of Genetics and Developmental Biology, Chinese Academy of Sciences, 286 Huaizhong Road, Shijiazhuang, Hebei 050021, China

**Keywords:** Lateral roots, osmotic stress, PEG 8000, premature differentiation, root tip, wheat (*Triticum aestivum*).

## Abstract

Water stress is one of the major environmental stresses causing growth retardation and yield loss of plants. In the past decades, osmotic adjustment, antioxidant protection, and stomatal movement have been extensively studied, but much less attention has been paid to the study of root system reprogramming to maximize water absorption and survival under water stress. Here, it is shown that polyethylene glycol (PEG)-simulated mild and moderate osmotic stress induced premature differentiation of the root apical meristem (RAM). It is demonstrated that RAM premature differentiation is a conserved adaptive mechanism that is widely adopted by various plants to cope with osmotic stress simulated by PEG 8000, and the occurrence of RAM premature differentiation is directly related to stress tolerance of plants. It is shown that the osmotic stress-induced premature differentiation caused growth cessation of primary roots allowing outgrowth of lateral roots. This work has uncovered a key mechanism for controlling the plastic development of the root system by which plants are capable of survival, growth, or reproduction under water stress.

## Summary

PEG-mediated osmotic stress induces premature differentiation of the root apical meristem, which in turn leads to outgrowth of lateral roots and improved tolerance to water stress.

## Introduction

Plant root growth is determined by cell elongation of the elongation zone and continuous production of cells of the apical meristem (Dinneny and Benfey, 2008). Root apices are established early during embryogenesis, and the root apical meristem (RAM), once formed in primary roots, maintains a proliferative state to generate radial and longitudinal tissues for the growing roots throughout the whole life of vascular plants (Ubeda-Tomas and Bennett, 2010). The RAM is composed of less mitotically active stem cells that surround the quiescent centre (QC) and the small cells with a high proliferative activity asymmetrically produced from one of the daughter cells of stem cells. These mitotically active cells divide and then the dividing cells in the basal region of the meristem start to differentiate triggered by unknown developmental signals entering the elongation zone (van den Berg *et al.*, 1995; Scheres, 2007; Dinneny and Benfey, 2008; Kornet and Scheres, 2009). Thus, the RAM maintains a stable size by balancing division of stem cells, and proliferation and differentiation of the meristematic cells under favorable conditions.

During development, however, plants encounter a variety of environmental stresses. Among them, water stress is the major abiotic stress affecting plant ecological distribution, crop growth, and productivity (Hsiao, 1973; Reddy *et al.*, 2004). Numerous studies have provided evidence to show that when plants are subjected to water stress, root growth is strongly inhibited, although root development is less sensitive to water stress than that of shoots (Westgate and Boyer, 1985; Sharp and Davies, 1989; Spollen *et al.*, 1993). Importantly, maintenance of root growth under water stress has been considered as an important adaptive trait for plants to increase deep water uptake and ensure their survival (Rodrigues *et al.*, 1995). Therefore, water stress perception and root responses of plants to water stress have been studied in maize (*Zea mays*) using a kinematic approach. It has been demonstrated that water stress is perceived by both the root tip and the apical region of the elongation zone, but not the basal region of the elongation zone and the mature region of maize primary roots (Sharp *et al.*, 1988). The spatial and temporal response patterns in growth rates of maize primary roots under water stress have also been revealed. When grown under well-watered conditions, the maximum elongation rate is detected in the region (4.5mm) of the elongation zone close to the root apex of the maize root. In contrast, in stressed plants, the elongation rate peaks in the region towards the apex, and then the relative elongation rate progressively lessens towards more basal regions (Sharp *et al.*, 1988, 2004). Thus, the apical region seems to play an important role in root elongation in response to water stress.

It has been proposed that elongation rates in the apical and basal regions of maize roots are regulated by different mechanisms (Sharp *et al.*, 2004; Shimazaki *et al.*, 2005). Physiological analyses have shown that proline accumulation dramatically increases several fold in the apical region (Voetberg and Sharp, 1991), suggesting that osmotic adjustment may contribute to growth maintenance in this region. Further studies showed that water stress-induced proline accumulation and the adaptive growth of the apical region of maize roots are dependent upon abscisic acid (ABA) (Voetberg and Sharp, 1991). However, genetic evidence indicates that ABA is not the only factor involved in elongation of the apical region of maize root in response to water stress. Corresponding to cell growth in the first few millimetres of the apical region (the meristematic region), enhanced cell wall loosening was detected in stressed roots (Wu *et al.*, 1996). Four expansin genes that regulate cell wall extension were specifically expressed in the apical growth region, supporting the observed cell growth in that region (Wu *et al.*, 2001). Genome-wide studies of expressed sequence tags (ESTs) and serial analysis of gene expression (SAGE) of different root tip sections with or without water stress have shown that ~20% of the transcriptome may be involved in water stress response of root cells and growth maintenance of the apical region of stressed maize roots (Sharp *et al.*, 2004). These findings suggest that water stress may alter root meristem activity resulting in root growth maintenance in the apical region and growth inhibition in more basal regions. However, to date, the mechanism for maintaining cell elongation in the apical region remains elusive.

Wheat (*Triticum aestivum*) is one of the most important grain crops, and water deficit is the most important constraint to wheat productivity worldwide. However, little is known about root responses to water stress. In a study to elucidate the mechanism of plant response in wheat, morphological responses of primary roots and plastic development of the root system in response to different levels of water stress stimulated by polyethylene glycol (PEG) 8000 were investigated. Intriguingly, it was found that the root tips of primary roots became dramatically enlarged, and anatomical analysis revealed that root tip swelling was caused by premature differentiation of the RAM rather than increased water absorption of RAM cells. Most importantly, further study demonstrated that root tip swelling is a universal adaptive mechanism of root responses to PEG-mediated mild and moderate water stress in plants, by which plants are able to enlarge the water absorption area by promoting the formation of extensive and long root hairs and stimulating lateral root development. Thus, this study reveals a key mechanism for controlling the plastic development of the root system by which plants are capable of survival, growth, or reproduction under PEG-mediated water stress.

## Materials and methods

### Plant materials and PEG 8000 treatments

Wheat seeds of Kn199, Sundor, SYN604, Xiaoyan-54, and Jing-411 were germinated and grown on filter papers wetted with water for 6 d under a 16h photoperiod at 22 °C, after which they were transferred to solutions containing different concentrations of PEG 8000 (PEG 8000/water, w/v) for 8h and 72h. *Arabidopsis thaliana* seedlings were germinated on Murashige and Skoog (MS) medium containing 1% sucrose (pH 5.7) for 5 d, and then treated with 5% PEG 8000 solution.

### Chemical treatments and microscope analysis

For Tetrazolium Violet staining, a 0.1% phosphate-buffered saline (PBS) stock solution was diluted 20 times and used for staining (Kurzbaum *et al.*, 2010). For lignin staining, the samples were mounted in staining solution (phloroglucinol in 20% HCl) and observed under a light microscope. Lignin appears red–violet in colour. For *Arabidopsi*s root observation, 5-day-old seedlings grown on MS medium were treated with 5% PEG 8000 solution for the indicated time, and the seedlings were then mounted in HCG solution (chloroacetaldehyde:water:glycerol 8:3:1). Images of the root meristem and QC zone were analysed using a Leica DM5000B microscope. For β-glucuronidase (GUS) staining, whole seedlings were immersed in the GUS staining solution (1mM X-glucuronide in 100mM sodium phosphate, pH 7.2, 0.5mM ferricyanide, 0.5mM ferrocyanide, and 0.1% Triton X-100), briefly subjected to a vacuum, and then incubated at 37 °C in the dark for at least 4h. The stained plant roots were photographed using a Leica DM5000B microscope.

### Paraffin sections

Paraffin sections were prepared as described by Chen *et al*. (2005). Briefly, root tips were excised (1cm segment) and fixed in FAA solution (formaldehyde:acetic acid:70% alcohol, 5:5:90) for 24h. Fixed tissues were washed with 50% ethanol for 30min. Dehydration was performed gradually with increasing concentrations of ethanol: 60, 75, 85, 95, and 100% ethanol for 1h each. For paraffin filtration, paraffin was added gradually to root materials at 65 °C for 3h. Roots were embedded in paraffin cakes, trimmed, and cut into sections of ~5 μm. Removal of paraffin and rehydration were started by adding 100% xylene for 15min, xylene:100% ethanol (1:1) for 15min twice, followed by a graded ethanol series (100, 95, 85, 70, and 50% ethanol) for 5min each. Root sections were then stained with safranin for 2h followed by two washes in 30% ethanol and sterile water, respectively. The safranin solution was replaced by Fast green FCF for a second staining for 1min before being washed twice in sterile water for 2min. Roots were then dehydrated in 30, 50, 70, 85, 95, and 100% ethanol for 3min each, followed by 3min in absolute ethanol:xylene (1:1), and 5min in 100% xylene, before being mounted in Euparal. All samples were photographed using a Leica DM5000B microscope.

### Microarray analysis and gene expression

Total RNA was extracted from the roots tips (1cm in length) of wheat seedlings using TRizol reagent according to the maufacturer’s manual (Tiangen Biotech). First-strand cDNA synthesis was performed using SuperScript^®^ II reverse transcriptase (Promega, 18064-014). Real-time PCR was performed as described in the manual. For microarray analysis, 6-day-old wheat plants were treated with 5% PEG 8000 solution for 0, 8, and 72h. Labelled cRNA samples were hybridized to an Affymetric GeneChip^®^ Wheat Genome Array containing 55 K wheat transcripts. Data from the GeneChip arrays were scanned on a GeneChip Scanner 3000 and analysed using GeneChip Operating software (GCOS 1.4). The Significant Analysis of Microarray (SAM) software was used to identify the genes differentially expressed in response to the treatments. The genes that were significantly differentially expressed were selected with a threshold of false discovery rate (FDR)=5% and fold change >1.5 in the SAM output result.

Real-time PCR was performed in 20 μl volumes containing 10 μl of 2× SYBR Premix Ex Taq mix (Takara), 0.2mM forward and 0.2mM reverse primers, 1 μl of diluted (1:10 v/v) first-strand cDNA, with a cycling regime comprising an initial denaturation step (95 °C/2min) followed by 40 cycles of 95 °C/5 s and 60 °C/34 s. A melting curve analysis was performed over the range 80–95 °C at 0.5 °C intervals. The wheat *actin* gene (GenBank accession no. AB181991.1) was used as a reference gene for the real-time PCR. All the experiments were repeated three times with three independent replicates each. Gene-specific primer sequences are listed in Supplementary Table S6 available at *JXB* online.

### Measurements of proline and soluble carbohydrates

Six-day-old wheat seedlings were treated in 5% PEG 8000 solution for 8h and 72h, and the root tips (1cm in length) were harvested for measurement of the proline content (Bates *et al.*, 1973). Soluble carbohydrates were extracted as described by Arthur Thomas (1977). All experiments were performed independently three times.

### Determination of histone modifications

The nuclei of wheat root tip cells treated with PEG 8000 were isolated as described by Zhu *et al.* (2008). A 20 μg aliquot of nuclear proteins was separated by SDS–PAGE and blotted onto a polyvinylidene difluoride (PVDF) membrane (Millipore, GVPPEAC12). Anti-tetra-acetylated histone H4 (1:1000), anti-histone H4 (1:100 000), anti-histone H3K4me2, anti-histone H3K27me3, anti-histone H3K9ac, anti-histone H3K9me2, and anti-histone H3 (Upstate Biotechnology) primary antibodies were used to detect acetylated and methylated histone H4 and histone H3; the bands were visualized using the BCIP/NBT Kit (Invitrogen). Data shown are representative of three independent experiments.

## Results

### PEG-mediated water stress induces premature differentiation of the RAM and reduction in root meristem size in wheat

**Fig. 1.**
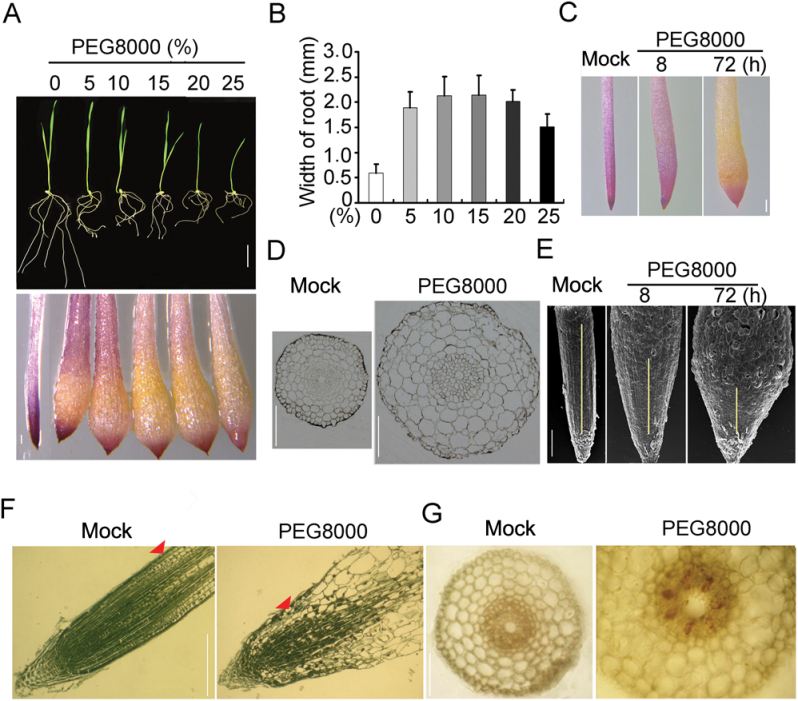
Effects of PEG 8000 treatments on 6-day-old wheat root tips. (A) Wheat seedlings (upper panel, scale bar=5cm) and root tips (lower panel, scale bar=0.5mm) were exposed to the indicated concentration of PEG 8000 solution for 72h. (B) Quantification of root tip width shown in (A). PEG 8000 at a 5% concentration was used for treatment at the indicated time in (C–G). (C) Tetrazolium Violet staining of wheat root tip. Scale bar=0.5mm. (D) Cross-sections of wheat root tip. Scale bar=0.4mm. (E) Scanning electron microscopy analysis of wheat root tip. Scale bar=0.5mm. (F) Longitudinal sections of root tip. The red arrows show the transition zone. Scale bar=0.5mm. (G) Phloroglucinol staining (reddish colour) of root cross-sections. Scale bar=0.2mm.

To understand the adaptive mechanisms associated with wheat root growth response to drought, a systematic analysis was conducted of root phenotypes using PEG 8000-simulated water stress in the Kn199 cultivar of wheat. When exposed to different levels of water stress for 72h (Supplementary Table S1 at *JXB* online), root growth of 6-day-old wheat seedlings was markedly reduced (Fig. 1A; Supplementary Fig. S1A–C at *JXB* online). Interestingly, it was found that the root tips of the stressed plants became swollen in the region which originally was the meristem zone (Fig. 1). Root tips treated with mild and moderate water stress (from –0.05MPa to –0.5MPa) displayed extreme swelling, whereas root tip swelling was less severe when wheat plants were treated with increasing levels of water stress (greater thaan –0.51MPa) (Fig. 1A, B).

To investigate the cellular mechanism of the morphological response of the root apex to water stress, a series of anatomical and histological examinations were performed. Because root tip swelling was a common response to mild and moderate water stress, further research thereafter was focused on analysis of the root tip response to mild stress (5% PEG 8000, –0.047MPa). It was found that the root tip swelling appeared starting from 8h after water stress at the transition region between the RAM and elongation zone, and then was aggravated toward the meristem zone over time, and reached the maximum size at 72h of treatment (Fig. 1C). Cross-section analysis of the most swollen section revealed that the size of the endodermis, stele, and, in particular, cortex cells of the swollen root tips was significantly increased, but the number of endodermis, stele, and cortical cell layers was not significantly changed compared with untreated roots (Fig. 1D; Supplementary Fig. S1D, E at *JXB* online). The result indicates that root tip swelling due to water stress is mainly caused by enlargement of cortex cells rather than by an increase in the number of cortex cells. Scanning electron microscopy analysis revealed that the epidermal cells of the swollen root section were prominent, larger, and more fragile compared with those of the non-stressed control root tips, so that the epidermis and outer cortical cells broke easily (Fig. 1E). In contrast, the epidermal cells closer to the root cap remained small and compact like the cells in the proximal meristem of the unstressed seedling root apex (Fig. 1E).

To test whether the root tip structure was altered by water stress, an analysis of longitudinal sections of the root tips including the meristem and elongation zones was conducted. Unexpectedly, the structure of root tips changed dramatically after exposure to water stress for 72h. The provascular tissues seemed to become differentiated in the enlarged sections of the stressed root tips during prolonged water stress (Fig. 1F). The primary vascular system extended to the stressed root tips and the size of the provascular tissue became very limited, which is in striking contrast to that of the non-stressed control (Fig. 1F). As a result, the size of the RAM was substantially reduced in the water-stressed wheat root tips compared with that of the control root tips (Supplementary Fig. S1F at *JXB* online). Phloroglucinol staining revealed that the cells of the xylem vessel in the swollen root tips were extensively lignified, and lignified vascular tissue extended from the differentiation zone to the root tip (Fig. 1G). Tetrazolium Violet staining, which is used for visualizing active cells (Kurzbaum *et al.*, 2010), showed that the meristem zone of the non-stressed roots was dark purple; in contrast, the swollen region of the stressed root tips became unstained and the darkly stained region was very limited, primarily to the root cap (Figs 1A, C, 3C). These findings demonstrate that PEG-mediated water stress induces premature differentiation of the RAM, resulting in reduced meristematic activity and eventual cessation of growth.

To confirm that the root tip swelling phenotype is caused by water deficit, root tips of wheat plants grown in pots and not watered were analysed. Three weeks after withholding watering when the relative water content (RWC) in soil gradually decreased from 80% to 20%, the root tips of the stressed seedlings were also swollen compared with those of control plants grown under well-watered conditions, although stressed roots had a milder phenotype (Supplementary Fig. S2 at *JXB* online). Enhanced accumulation of lignin in the vascular vessels of the swollen root tips was also observed. These data suggest that this particular root developmental phenotype, namely root tip swelling and premature differentiation of the RAM, may be a general response adopted by plants in response to water deficit.

### Osmolytes, ROS, and compatible soluble compounds are involved in premature differentiation of the RAM of wheat under water stress

**Fig. 2.**
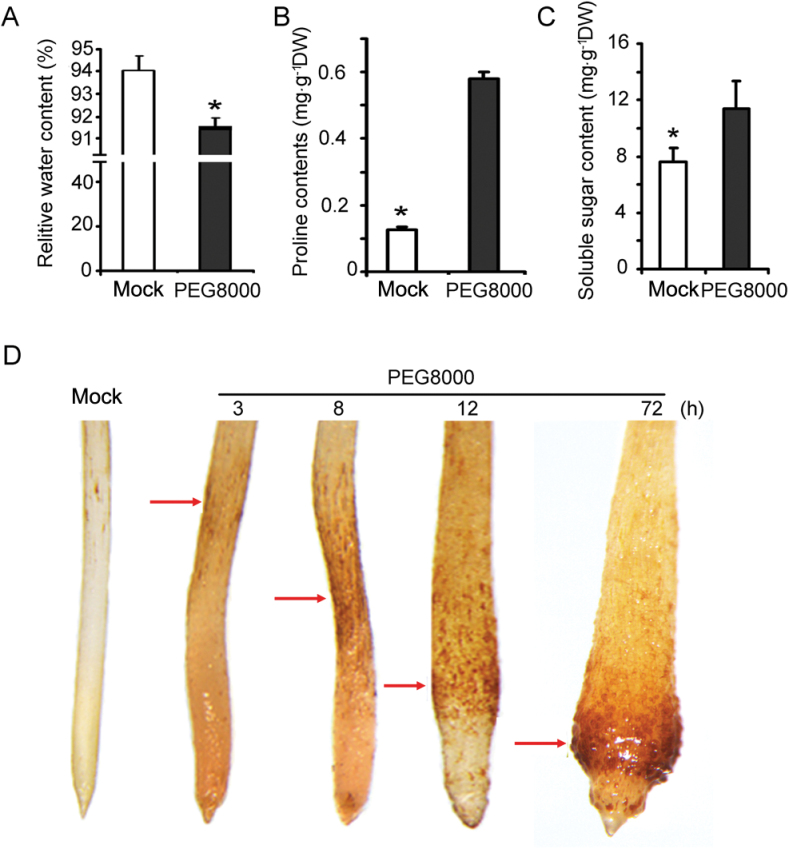
Physiological analysis of wheat RAM. (A) Relative water content (RWC); (B) proline content (mg g^–1^ DW); (C) soluble sugar content (mg g^–1^ DW). Data are presented as means ±SD, and columns marked with asterisks indicate significant differences in each treatment using Student’s *t*-test analysis (*P*<0.05). Experiments were repeated three times with similar results. (D) Detection of hydrogen peroxide by DAB (3,3’-diaminobenzidine) staining in the root tip at the indicated time point.

Since the cells of the prematurely differentiated RAM were extremely enlarged, it was questioned whether the cells absorb excessive water to help them survive in dry soil. To determine whether this was the case, physiological analyses were performed to investigate the significance of the root tip swelling and differentiation of the RAM under water stress. The same sizes (1cm in length) of root tips of stressed and control plants were collected and examined at 72h after treatment. The RWC of the root samples with or without stress treatment was measured. Unexpectedly, the RWC of the swollen root tips (91.97%) induced by water stress was significantly lower than that of the untreated control (93.91%) (Fig. 2A), suggesting involvement of osmotic adjustment of the prematurely swollen root tips for adaptation to PEG-mediated water stress.

Because proline is an important osmolyte that accumulates during water stress to help maintain cell turgor and growth (Voetberg and Sharp, 1991; Verslues and Sharp, 1999), the levels of proline in the stressed and non-stressed root tips were measured. As expected, the proline level of the swollen root tips was dramatically increased under water stress. The proline content of the stressed root tips at 72h after stress was ~5-fold higher than that of the non-stressed control (Fig. 2B). The content of soluble sugars, another indicator of osmotic adjustment, was also markedly increased in the stressed root tips compared with those of the unstressed control (Fig. 2C). This result indicates that swelling and premature differentiation of root tips may be an adaptive morphological response to water stress simulated by PEG 8000.

Previous studies in maize have suggested an important role for reactive oxygen species (ROS) in root tip growth under water stress (Sharp *et al.*, 1994; Ober and Sharp, 2003). To test whether ROS are involved in wheat meristem premature differentiation of stressed root tips, the accumulation of H_2_O_2_ in root tips was examined using 3,3’-diaminobenzidine (DAB) staining. Under normal conditions, H_2_O_2_ was hardly detected in the control roots including the root tips (Fig. 2D). However, H_2_O_2_ accumulation was markedly increased in the stressed root tip region at 3h after treatment. Interestingly, accumulation of H_2_O_2_ in the root tip region was not uniform. At 3h after treatment, a greater accumulation of H_2_O_2_ was detected in the elongation region, and H_2_O_2_ accumulation was more pronounced and its distribution area was enlarged throughout the elongation region at 8h after treatment. With prolonged water stress, greater accumulation of H_2_O_2_ was detected in the enlarged region toward the meristem zone of the stressed root tips (Fig. 2D). This is in sharp contrast to low H_2_O_2_ accumulation in the cells proximal to the root cap. H_2_O_2_ accumulation in the meristem zone of the stressed roots reached a maximum when the apical meristem of the stressed root was at its largest at 72h after treatment (Fig. 2D). It is apparent that the pattern of H_2_O_2_ accumulation during water stress is correlated to the progression of root tip premature differentiation, suggesting a critical role for H_2_O_2_ in root tip swelling and premature differentiation of the RAM in response to PEG-simulated water stress in wheat.

### Water stress-induced premature differentiation of the RAM causes growth cessation of the primary root, and lateral root development

**Fig. 3.**
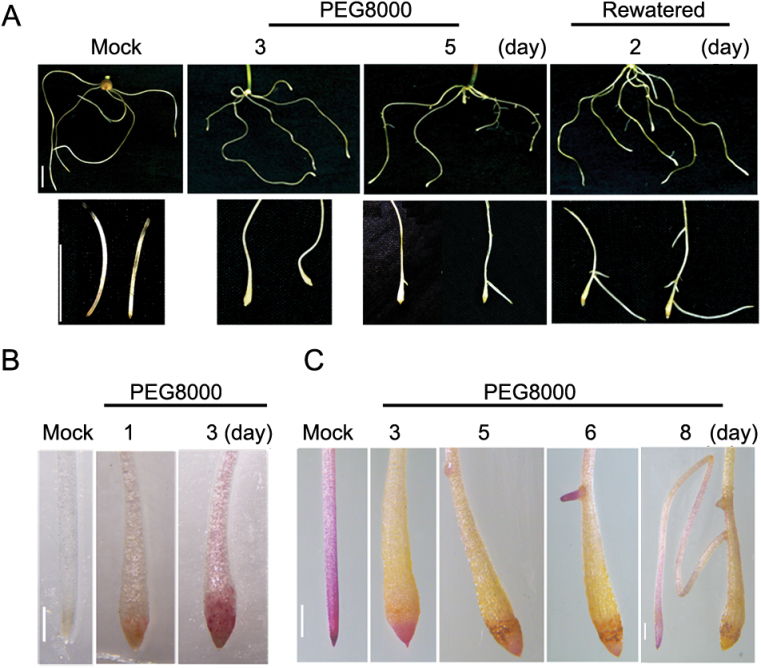
Root tip swelling causes cessation of primary root growth and promotes lateral root development. (A) Image of the whole root system (upper panel) under 5% PEG 8000 treatment for 0 (Mock), 3 (3), and 5 d (5), and removal of water stress for 2 d (Rewatered); the corresponding growth of root tips is shown in the lower panel. Scale bar=2cm. (B) Phloroglucinol staining (reddish colour) of the root tips with or without PEG 8000 treatment. Scale bar=0.5mm. (C) Tetrazolium Violet staining of wheat roots with or without 5% PEG 8000 treatment. Scale bar=0.5mm.

It was previously reported that severe water stress-induced programmed cell death (PCD) of meristematic cells in the root tips causes root growth cessation resulting in induction of lateral root development in *Arabidopsis* (Duan *et al.*, 2010). Thus, it was speculated that premature differentiation of the RAM of the wheat roots under PEG-induced water stress may be another adaptive mechanism for plants to cope with mild and moderate water stress. To this end, growth and development of the root system under prolonged water stress conditions were investigated. It was found that the swollen root tips did not grow under prolonged water stress or after removal of the stress (Fig. 3A). Trypan blue staining showed that the cells of the swollen roots were still alive (Supplementary Fig. S3 at *JXB* online), suggesting that growth cessation of the swollen roots induced by PEG-mediated water stress is caused by premature differentiation of the root meristem and elongation regions. Notably, when exposed to prolonged water stress, the stressed roots gradually became lignified, especially some cells in the swollen region of the stressed roots (Fig. 3B). Lateral roots started to appear in the region originally occupied by the mature zone at 5 d after water stress treatment, and continued to grow under water stress conditions (Fig. 3A, C). These lateral roots were capable of maintaining normal growth when stressed plants were transferred to normal growth conditions (Fig. 3A). These results demonstrate that premature differentiation of the RAM is an important adaptive mechanism for plant roots to reprogramme root system development in response to mild and moderate water stress.

### The premature differentiation of the RAM induced by water stress is correlated with plant stress tolerance

**Fig. 4.**
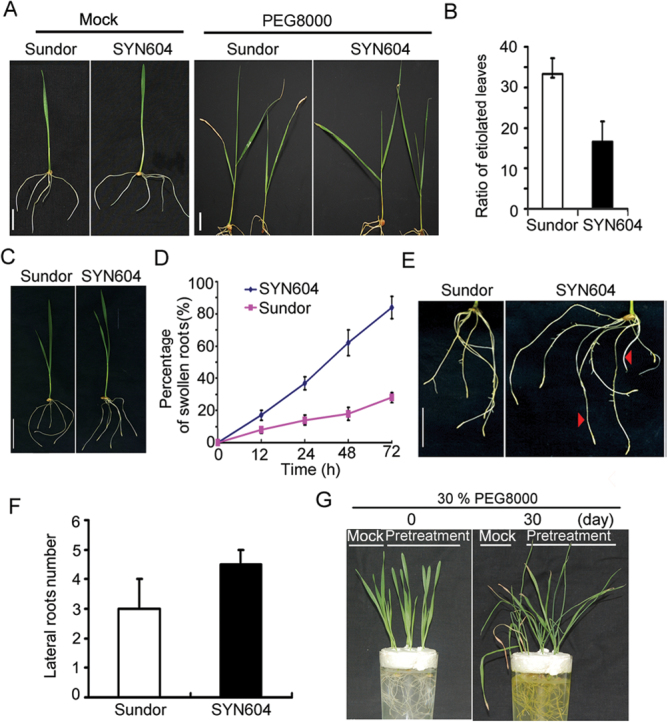
The premature differentiation of root tips is correlated with plant drought tolerance. Scale bar=2cm. In (A–F) 5% PEG 8000 was used. (A) Six-day-old seedlings (Mock) were transferred to PEG 8000 for 30 d (PEG 8000). (B) Comparison of the ratio of etiolated leaves of Sundor and SYN604. (C) Sundor and SYN604 were subjected to PEG 8000 treatment for 3 d. (D) Comparison of the percentage of swollen roots between Sundor and SYN604 at the indicated time point under PEG 8000 treatment. (E) Roots of Sundor and SYN604 under PEG 8000 treatment for 10 d. Arrowheads show the crown roots. (F) Quantification of the lateral root number of Sundor and SYN604 shown in (E). (G) Six-day-old seedlings were pre-treated (Pretreatment) or not (Mock) with 5% PEG 8000 for 3 d, and then exposed to 30% PEG 8000 treatment for 30 d. (This figure is available in colour at *JXB* online.)

To understand the biological role of the premature differentiation of the RAM under mild and moderate water stress induced by PEG 8000, experiments were performed to test the effects of the stress-induced swollen root tips on plant tolerance to water stress. Root tip swelling of a pair of wheat cultivars with different sensitivity to drought was analysed under mild water stress. As shown in Fig. 4A and B, SYN604 seedlings were more tolerant to drought than Sundor seedlings, which is consistent with a previous report (Ji *et al.*, 2010). Root tip growth of both cultivars was quite normal when grown under normal conditions (Fig. 4A). When the wheat seedlings were subjected to mild water stress, root tip swelling was also observed (Fig. 4C). Interestingly, root tip swelling of SYN604 appeared much earlier than that of Sundor when treated with mild water stress (Fig. 4D). There was an extremely high number of swollen root tips in stressed SYN604. Nearly 82% of SYN604 root tips were swollen at 72h after water stress, whereas <25% of Sundor root tips had visible swelling (Fig. 4D). The result suggests that root tip swelling may be directly related to drought sensitivity of wheat seedlings. When the root systems of the two cultivars were compared, it was found that the average root lengths of the two varieties were not significantly different, but SYN604 had more lateral roots than Sundor (Fig. 4E, F). Notably, several crown roots at a time occurred in the stressed SYN604 roots, whereas none was observed in Sundor roots (Fig. 4E). Another two different genotypes of wheat, Xiaoyan-54 (high drought resistance) and Jing-411(susceptible to drought) (Sečenji M, *et al*., 2010*a*, *b*), were used to study the effects of PEG 8000 treatment. Swollen root tips of Xiaoyan-54 appeared much earlier than those of Jing-411 when treated with mild water stress (Supplementary Fig. S4A, B at *JXB* online), and Xiaoyan-54 had longer lateral roots than Jing-411 (Supplementary Fig. S4C at *JXB* online); however, Xiaoyan-54 had more lateral roots than Jing-411 (Supplementary Fig. S4D at *JXB* online). These results suggest that root tip premature differentiation may be directly related to drought sensitivity of wheat seedlings.

To verify the correlation between root tip premature differentiation and adaptation to drought, the effect of pre-treatment of wheat seedlings with 5% PEG 8000 on the drought tolerance to a high level of water stress was also tested. Seedlings treated or not with 5% PEG 8000 for 3 d were transferred to growth medium containing 30% PEG 8000 for 30 d, and the stress responses of the plants were observed. As shown in Fig. 4G, the plants without pre-treatment showed more severe symptoms than the pre-treated plants with swollen root tips. The leaf tips of all control plants were brown and dead; in contrast, fewer leaf tips were dead in the pre-treated plants. Accordingly, the pre-treated plants had more and longer lateral roots than the control plants (data not shown). Taken together, these results demonstrate that rapid root tip swelling and growth cessation may be an adaptive mechanism to facilitate lateral root development under water stress to enlarge the water absorption area and confer enhanced ability to survive under water stress.

### A number of genes and histone modifications are involved in premature differentiation of the RAM and root responses to water stress

**Fig. 5.**
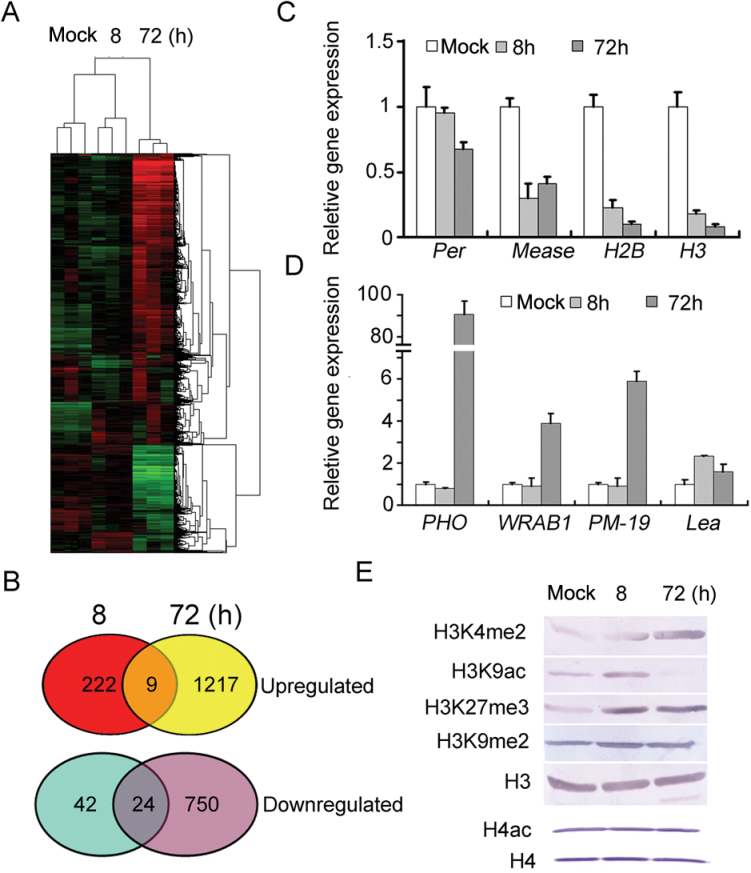
Changes in gene expression and histone modifications in the RAM under 5% PEG 8000 treatment. (A) Hierarchical clustering analysis of the differentially expressed genes in wheat root tips treated with PEG 8000 at 0 (Mock), 8 (8), and 72 (72) h. Changes in gene expression are displayed from blue (down-regulated) to red (up-regulated). There were three biological replicates for each time point. (B) Venn diagrams showing up-regulated genes (upper panel) divided into the common genes (overlap region) parts and unique differentially expressed genes under 8h (red) and 72h (yellow) PEG 8000 treatment; the lower Venn diagrams show down-regulated genes divided into the common genes (overlap region) and unique differentially expressed genes under 8h (blue) and 72h (purple) PEG 8000 treatment. Real-time PCR analysis of the differentially expressed genes; four down-regulated genes (C) and four up-regulated genes (D) were tested. Wheat *actin* was used as an internal reference. There were three biological replicates for each time point. (E) H3 but not H4 modifications in wheat root were changed with PEG 8000 treatment. Equal amounts of total histones extracted from wheat root with or without PEG 8000 were immunoblotted with the indicated antibodies. (This figure is available in colour at *JXB* online.)

To investigate the molecular mechanism of root tip swelling under drought conditions, root tips (1cm length from the root tip) of the Kn199 variety of wheat were collected at 0, 8, and 72h after mild water stress in order to analyse the gene transcriptional profiles. The root tips of seedlings grown under normal conditions were used as the control. Cluster analysis of microarray data showed that many genes were up-regulated or down-regulated after water stress and the number of genes with altered expression was substantially increased during a prolonged period of water stress (Fig. 5A). In total, 297 unigenes were up-regulated (231 ≥2.0-fold) and 66 genes were down-regulated (≤0.5 fold) at 8h after treatment. In contrast, at 72h after treatment, 2000 differentially expressed unigenes (1226 genes up-regulated ≥2-fold and 774 genes down-regulated ≤0.5-fold) were detected. In total, eight genes were up-regulated at both 8h and 72h after treatment, while 24 genes showed decreased expression at both time points (Fig. 5B; Supplementary Table S2 at *JXB* online). There were 107 pathways and 388 pathways (in comparison with rice mRNA data) involved in response to drought stress at 8h and 72h, respectively (Supplementary Table S3 at *JXB* online). Gene ontology (GO) analysis revealed that expression of many mitochondrial, cytoplasmic membrane-bound vesicle-, cytoplasm-, cell wall-, and plastid-related genes changed under drought stress (Supplementary Tables S4, S5 at *JXB* online). Many genes that are involved in hormone biosynthesis and signalling and light response were also differentially expressed in response to water stress. In particular, many genes related to histone modification, ROS metabolism, and ABA were also differentially expressed under water stress (Supplementary Table S2 at *JXB* online). The result indicates that root tip swelling of wheat seedlings under water stress is a complex process that is regulated by a complex molecular network.

To verify the microarray data, quantitative real-time PCR analysis was used for experimental validation of the gene expression patterns of the root tips with or without water stress. The results showed that the expression levels of *LEA* (embryo-specific protein 1, Ta.9830.1.A1_at), *PHO* (photosystem II polypeptide, Ta.27793.2.S1_x_at), *WRAB* (*Triticum aestivum* ABA-inducible protein, Ta.226.1.S1_at), and *PM-19* (*Triticum aestivum* ABA-induced plasma membrane protein, Ta.129.1.S1_at) genes were substantially increased at 72h after treatment, whereas expression of *Per* (peroxidase, Ta.21137.1.S1_x_at), *histone H3* (histone H3 variant, Ta.24759.1.S1_at), *histone H2B* (histone H2B variant, Ta.7378.18.S1_x_at), and *Mease* (*O*-methyltransferase, Ta.23042.2.S1_at) genes was down-regulated under drought stress (Fig. 5C, D). The expression patterns of the tested genes are consistent with those in the microarray data, confirming involvement of these genes and pathways in root tip swelling under water stress.

Alterations in histone and histone methyltransferase expression prompted the investigation of whether histone modifications are involved in transcriptional regulation of genes modulating water stress-induced root tip swelling in wheat. To this end, the levels of dimethylation of Lys4 on histone H3 and acetylation at Lys9 on histone H3, which are associated with gene activation, as well as the levels of dimethylation of Lys9 on histone H3 and trimethylation of histone H3 at Lys27, which have been shown to correlate with gene silencing (Bernstein *et al.*, 2006), were analysed. As shown in Fig. 5E, dimethylation of Lys4 on histone H3 (H3K4me2) was markedly increased at 8h after water stress and reached the highest level at 72h after water stress. A global increase in the H3K9AC level was also detected at 8h after treatment, but a large reduction in H3K9AC was observed with prolonged exposure to water stress. The level of H3K27me3 trimethylation was also rapidly increased under drought stress, and dimethylation of Lys9 on histone H3 was slightly increased. The level of global histone H4 tetra-acetylation at K5/K8/K12/K16, which is also associated with gene activation, was also examined under water stress. Unexpectedly, the level of tetra-acetylated histone H4 was not significantly changed under drought stress (Fig. 5E). These results indicate that histone H3 modification may play a more important role in modulating gene expression and premature differentiation of root tips in response to water stress.

### RAM premature differentiation induced by PEG-mediated water stress was widely adopted in plants

**Fig. 6.**
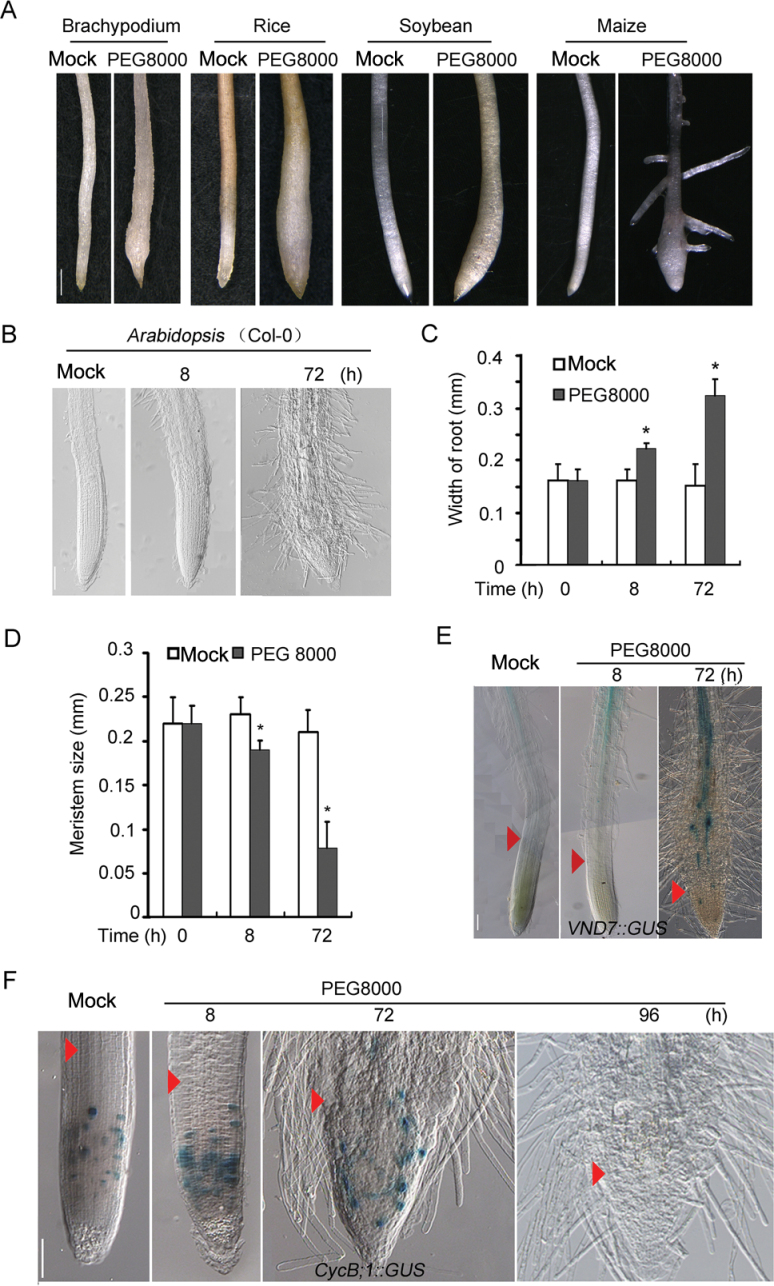
Swollen root tips induced by water stress were widely adopted in higher plants. (A) Ten-day-old *Brachypodium distachyon* (*Brachypodium*), rice, soybean, and maize were treated with 5% PEG 8000 for 3, 3, 5, and 10 d, respectively. Scale bar=1mm. (B) Five-day-old *Arabidopsis* (Col-0) seedlings were treated or not with PEG 8000 for 8h or 72h. Scale bar=0.1mm. (C) Quantification of root width shown in (B). (D) Quantification of root meristem size shown in (B). (E) Expression analysis of *VND7* using *VND7::GUS* transgenic plants with or without 5% PEG 8000. Arrowheads indicate the cortex transition zone. Scale bar=0.1mm. (F) Expression analysis of *CycB;1* using *CycB;1::GUS* transgenic plants with or without 5% PEG 8000. Arrowheads indicate the cortex transition zone. Scale bar=0.1mm.

To examine whether RAM premature differentiation under PEG-mediated water stress is a universal phenomenon in higher plants, the root stress responses of *Arabidopsis* and several crop species were tested. It was found that the root tips of all tested plant species were swollen when they were subjected to water stress (Fig. 6A). Among them, *Brachypodium distachyon*, a relative species of wheat, rice, and *Arabidopsis*, showed a quite similar swollen root tip phenotype to wheat under 5% PEG 8000 treatment, whereas other plants including maize and soybean treated with the same level of water stress displayed a milder swollen root tip phenotype (Fig. 6A, B).

To confirm the cellular mechanism of the root tip response to water stress, meristematic activity, meristem sizes, and premature differentiation of the root tips in *Arabidopsis* were examined under water stress. As shown in Fig. 6B, the root tips of the stressed seedlings were enlarged at 8h after treatment, and became swollen at 72h after stress. Correspondingly, the meristem sizes of the stressed root tips became smaller than those of the control, as shown both by the first enlarged cortex cell above the meristem, which is usually used as an indicator of the border between the meristem and elongation zone, as well as the appearance of root hairs, which is an indication of the mature zone of roots (Fig. 6C, D). Staining analysis of *CycB1;1::GUS* showed that *CycB1;1* expression was increased in a smaller region close to the root cap, and the number of cells expressing *CycB1;1* decreased with prolonged water stress. No *CycB1;1* expression was detected at 96h after water stress, indicating that cells of root tips have lost their division activity (Fig. 6F). Further analysis of the expression pattern of *VND*7, a marker for vascular differentiation (Yamaguchi *et al*., 2008), under water stress also clearly showed differentiation in root tips (Fig. 6E), which is consistent with the observation in wheat (Figs 1F, G, 3B).

## Discussion

Water is essential for survival and plant growth. As a sessile organism, plants constantly encounter water deficit, which is the most severe adverse environmental stress limiting plant growth and productivity in natural and agricultural systems (Hsiao, 1973; Reddy *et al.*, 2004). Thus, water stress tolerance has been a fundamental scientific question in plant biology. Plants have evolved complex adaptive mechanisms that enable them to survive drought conditions. Over more than five decades, researches have identified osmotic adjustment, antioxidant protection, and stomatal movement as key adaptive mechanisms for survival and growth in growth conditions lacking sufficient water (Sharp *et al.*, 1990; Bailey-Serres and Mittler, 2006; Yamaguchi *et al.*, 2010). However, developmental/morphological responses of plants and the underlying mechanisms in response to water deficit have not attracted much attention. A previous study has demonstrated that severe water deficit induces PCD of the RAM to promote development of lateral roots with enhanced drought tolerance (Duan *et al.*, 2010). In natural growth environments, plants often need to cope with mild and moderate levels of water deficit. However, to date, how plant roots respond to mild and moderate water stress and what mechanisms underly the stress responses of roots remain elusive. The present results indicate that low levels of water stress simulated by PEG 8000 induce premature differentiation of the RAM in plants, and both osmotic adjustment and ROS are involved in this plastic development process. Evidence is provided that water deficit-induced premature differentiation of RAM is an active adaptive mechanism for plants in response to mild and moderate water stress simulated by PEG 8000. Thus, a key developmental mechanism in plants to adapt to mild and moderate water stress is explained for the first time,

### PEG-mediated mild and moderate water stress promotes premature differentiation of the RAM

Plants absorb water from soil mainly through the surface of young roots containing extensive root hairs. It has long been known that plant roots have a unique feature of indeterminate growth throughout their life, because they have the RAM for primary growth in all roots including primary roots and lateral roots. Thus, plants can continuously generate young roots to absorb water when plants grow in an environment containing enough water. However, little is known about how plant roots respond to water stress and maximize water absorption; in particular, evidence of the root morphological adaptation to mild and moderate water stress and the underlying mechanisms has been lacking.

In this study, it was found that primary root tips of wheat seedlings were swollen (Fig. 1). Further analysis of the model plants *B. distachyon* and *Arabidopsis*, and important crops including soybean, rice, and maize (Fig. 6A, B) demonstrated that root swelling was a general morphological response in both monocot and dicot plants to PEG-mediated mild and moderate water stress. The data obtained from potted wheat plants under progressive water stress validated the hypothesis. However, the degree of root tip swelling varies a great deal from species to species in response to the same level of water stress (data not shown), and the growth medium can also affect the degree of root tip swelling tremendously. For example, the root tips of stressed plants grown in soil showed a lesser degree of root swelling than those of plants cultured in liquid medium (Fig. 1; Supplementary Fig. S2 at *JXB* online). In addition, the varying levels of root tip enlargement and the time of occurrence of root tip swelling under the same level of water stress may also be related to the sensitivity of a plant to drought (Fig. 4; Supplementary Fig. S4 at *JXB* online). This may be the reason for the inconsistent reports on the root tip response to water stress in different species (Sharp *et al.*, 1988; Vartanian *et al.*, 1994; Chen *et al.*, 2006).

It was then questioned whether the root tip swelling is a mechanism for plant roots to absorb more water, protecting root meristem cells from water stress. The fact that the swollen root tips contained less water (Fig. 2A) and increased levels of proline and soluble sugars (Fig. 2B, C) supports the notion that swollen root tips have a high osmotic adjustment rather than more water. Intriguingly, the RAM became differentiated under water stress (Figs 1F, G, 3B, 6E), accompanied by enlargement of newly differentiated cortex cells (Fig. 1D, G) and a reduced RAM. When exposed to prolonged water stress, the RAM was totally differentiated (Figs 1F, 6D, E; Supplementary Fig. S1F at *JXB* online) resulting in cessation of growth of the stressed roots. Thus, the findings reveal a novel mechanism for plants to cope with PEG-mediated mild and moderate water stress that is apparently different from the growth cessation of roots caused by PCD induced by severe water stress.

### RAM premature differentiation enhances plant tolerance to PEG-mediated osmotic stress

The next question is whether RAM premature differentiation is associated with plant tolerance to PEG-mediated osmotic stress. As mentioned earlier, young roots with root hairs are responsible for water absorption. Thus the surface area of young roots and root hairs largely determine the water absorption. Therefore, an increase in surface area of young roots should be an adaptive strategy for plants to absorb more water. It is noteworthy that in addition to root tip swelling, extensive and long root hairs were induced in the newly differentiated region under water stress (Fig. 6B–F). It is conceivable that both water stress-induced root tip swelling and root hair development substantially increased the root surface area for water absorption, leading to an improved ability for plant root cells to cope with water stress. Therefore, it is concluded that water stress-induced RAM premature differentiation is an adaptive developmental response to water stress in plants.

PCD-mediated loss of function in meristematic activity of roots stimulates emergence of lateral roots (Duan *et al.*, 2010). It is proposed that premature differentiation of the RAM under PEG-mediated mild and moderate water stress may have a similar function in removing root apical dominance and promoting lateral root growth. Indeed, cessation of growth of the primary roots because of premature differentiation of the RAM under PEG-mediated water stress promotes lateral root emergence at the sites above the swollen regions of the primary roots during prolonged water stress (Figs 3A, 4E). It is apparent that continuous emergence and growth of these newly formed lateral roots under water stress enlarges the root surface area for water absorption. These observations imply that premature differentiation of the RAM is a critical morphological response related to stress tolerance. Four lines of evidence support the morphological phenotype linkage to plant tolerance to PEG-mediated water stress. First, a drought-tolerant cultivar showed much earlier occurrence of RAM differentiation and a significantly higher rate of RAM premature differentiation at 72h after treatment than a drought-susceptible cultivar (Fig. 4C, D; Supplementary Fig. S4 at *JXB* online). This finding proves that RAM premature differentiation is directly associated with stress tolerance in plants. Root tip swelling may be used as an indicator for evaluating plant drought tolerance. Secondly, the plants with differentiated RAMs pre-treated with low levels of water stress showed enhanced stress tolerance compared with the non-treated control when exposed to an elevated level of water stress (Fig. 4G). The results suggest that reprogramming occurs within hours of water stress, resulting in an increased ability of plants to acclimate to increasing levels of stress conditions and alteration in tolerance to PEG-mediated water stress. Genome-wide expression analysis of wheat root tips (Fig. 5A; Supplementary Table S2 at *JXB* online) supports transcriptional regulation of the genes related to drought tolerance in wheat. Altered expression of many genes related to ABA, ROS homeostasis, osmotic adjustment, histone functions, and cell expansion suggest that premature differentiation of the RAM is a highly complex process and an integrated network involving antioxidant and redox regulation, metabolism control, and hormonal regulation of cell response and plastic development. Global changes in H3K4me2, H3K9ac, and H3K27me3 (Fig. 5E) confirm genome-scale epigenetic reprogramming during RAM premature differentiation in response to water stress. Thus, the evidence leaves no doubt about the important role of RAM premature differentiation in drought tolerance.

In summary, it was demonstrated that RAM premature differentiation is an adaptive mechanism that is widely adopted by plants in response to PEG-mediated mild and moderate water stress. It is intriguing to speculate that activation of RAM premature differentiation is in fact more complex. Thus, although the mode of perception of water stress and signalling transduction to activate root growth determination remains unknown, a key mechanism for controlling the plastic development of root system and stress response by which plants cope with environmental stress has been revealed.

## Supplementary data

Supplementary data are available at *JXB* online.

Figure S1. Effects of PEG 8000 treatments on wheat root tips.

Figure S2. PEG-mediated water deficit-induced root swelling and premature differentiation.

Figure S3. Epidermal cells in the swollen root region remain alive.

Figure S4. Swollen roots appeared earlier in Xiaoyan-54 than in Jing-411 under PEG-mediated water stress.

Table S1. Evaluation of the water potentials of PEG 8000 solutions.

Table S2. Differential gene expression under PEG 8000 treatment in wheat root tip.

Table S3. Pathway analysis of the differentially expressed genes.

Table S4. Cellular component analysis of the differentially expressed genes.

Table S5. Biological function analysis of the differentially expressed genes.

Table S6. Primers used for real-time PCR.

Supplementary Data
