# Supplementary material for: PEG-mediated osmotic stress induces premature differentiation of the root apical meristem and outgrowth of lateral roots in wheat
Source: J Exp Bot. 2014 Jun 16;65(17):4863–72. doi: 10.1093/jxb/eru255 (PMC4144773; doi:10.1093/jxb/eru255)
Supplement: Supplementary Data [file supp_65_17_4863__index.html]

PEG-mediated osmotic stress induces premature differentiation of the root apical meristem and outgrowth of lateral roots in wheat — PEG-mediated osmotic stress induces premature differentiation of the root apical meristem and outgrowth of lateral roots in wheat — Supplementary Data 

# PEG-mediated osmotic stress induces premature differentiation of the root apical meristem and outgrowth of lateral roots in wheat

## Supplementary Data

Data files

**Files in this Data Supplement:**

- Supplementary Data - Supplementary Data
- Supplementary Data - Supplementary Data
- Supplementary Data - Supplementary Data
