# Supplementary material for: PEG-mediated osmotic stress induces premature differentiation of the root apical meristem and outgrowth of lateral roots in wheat
Source: J Exp Bot. 2014 Jun 16;65(17):4863–72. doi: 10.1093/jxb/eru255 (PMC4144773; doi:10.1093/jxb/eru255)
Supplement: Supplementary Data [file supp_eru255_jexbot118281_file001.pdf]

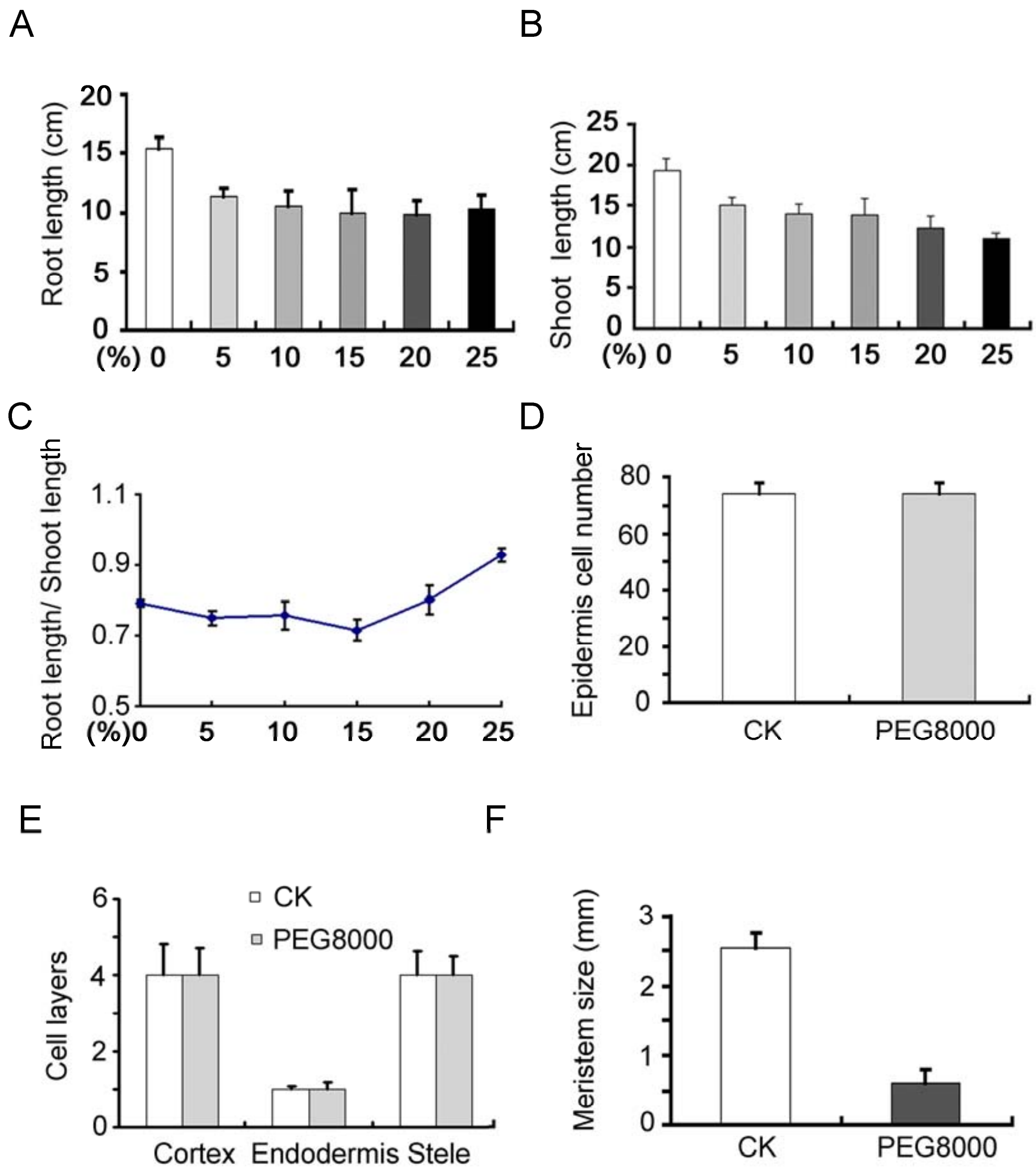

Supplementary figure S1. Effects of PEG 8000 treatments on wheat root tips. A, Quantification of root length shown in Fig. 1A. B, Quantification of shoot length shown in Fig. 1A. C, Ratio of root length/shoot length shown in Fig. 1A. D, Quantification of epidermis cell number shown in Fig. 1D. E, Quantification of epidermis, cortex and stele cell layers shown in Fig. 1D. F, Quantification of meristem size shown in Fig. 1F.

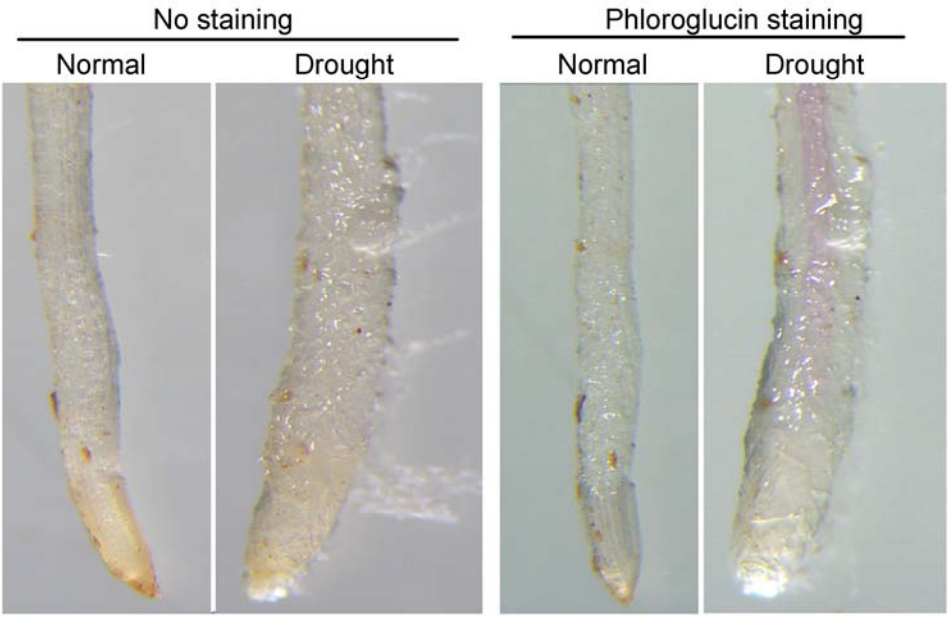

Supplementary figure S2. PEG-mediated water deficit induced root swollen and premature differentiation. Phloroglucinol staining of lignin (reddish color) of the root tips which were responses to drought stress (20% of soil water-holding capacity) and Normal (80% of soil water-holding capacity) for three weeks.

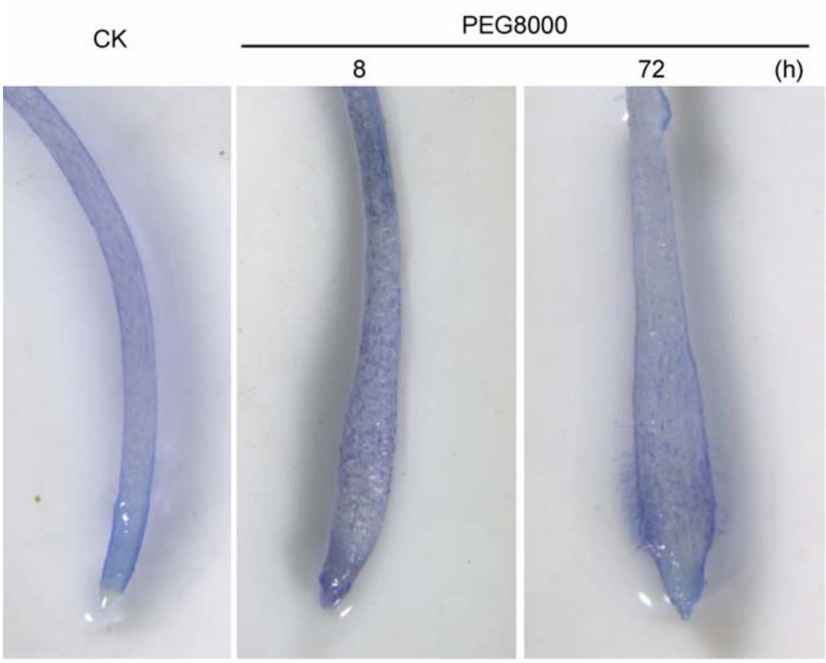

Supplementary figure S3. Epidermal cells in swollen root region remain alive. Typan blue staining of 5% PEG 8000 treatment root samples at indicated time.

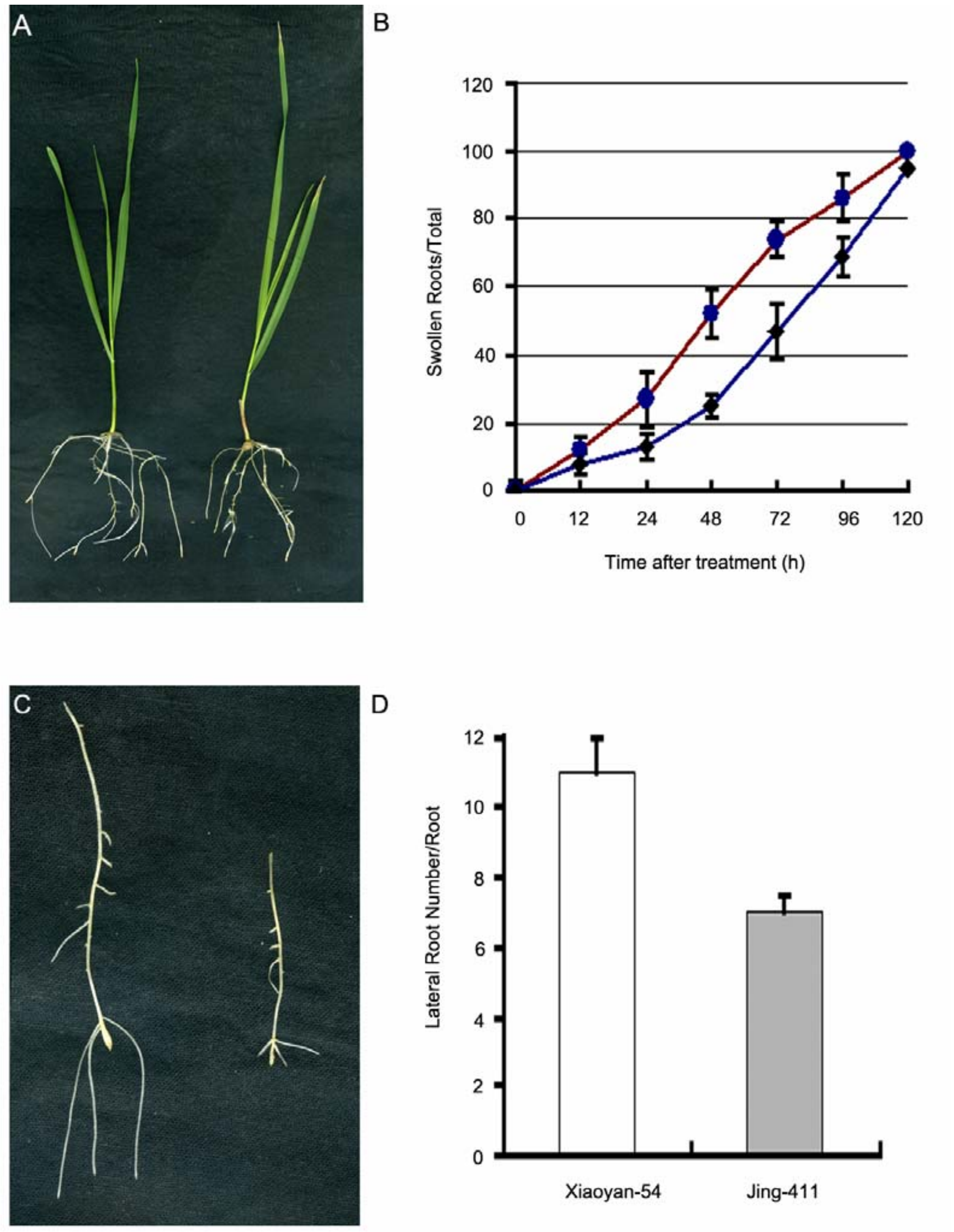

Supplementary figure S4. Xiaoyan-54 appeared earlier swollen roots than that of Jing-411 under PEG-mediated water stress.

A, six-day-old Xiaoyan-54 (left) and Jing-411 (right) were treated in 5% PEG 8000 solution for 5 days.

B, Quantification of percentage of swollen roots between Xiaoyan-54 and Jing-411 at indicated time point under PEG 8000 treatment.

C, Xiaoyan-54 (left) showed longer lateral root than that of Jing-411 (right) under water stress.

D, Quantification of lateral root number of Xiaoyan-54 and Jing-411shown in (A).

Supplementary Table S1. Evaluation of the water potentials of PEG 8000 solutions.

Water Potential (MPa)	PEG 8000 Concentration (w/v)	Temperature (°C)
-0.0469	5 %	25
-0.14775	10 %	25
-0.302	15 %	25
-0.511	20 %	25
-0.573	25 %	25
-1.08975	30 %	25

Supplementary Table S4. Cellular component analysis of the differentially expressed genes (only GO categories with 20 or more genes are shown).

Rank	Cellular Component (CK vs 8 h)	No. of genes	Cellular Component (CK vs 72 h)	No. of genes
1	GO:0005739 mitochondrion	152	GO:0005739 mitochondrion	673
2	GO:0016020 membrane	107	GO:0009536 plastid	598
3	GO:0016023 cytoplasmic membrane-bound vesicle	90	GO:0016020 membrane	427
4	GO:0005634 nucleus	77	GO:0016023 cytoplasmic membrane-bound vesicle	411
5	GO:0005737 cytoplasm	64	GO:0005634 nucleus	322
6	GO:0005618 cell wall	51	GO:0005737 cytoplasm	265
7	GO:0005622 intracellular	22	GO:0005618 cell wall	248

Supplementary Table S5. Biological functions analysis of the differentially expressed genes (only GO categories with 30 or more genes are shown).

Rank	Biological Function (CK vs 8 h)	No. of genes	Biological Function (CK vs 72 h)	No. of genes
1	GO:0009987 cellular process	68	GO:0009719 response to endogenous stimulus	271
2	GO:0006950 response to stress	62	GO:0009987 cellular process	257
3	GO:0007582 physiological process	56	GO:0006950 response to stress	241
4	GO:0009719 response to endogenous stimulus	55	GO:0007582 physiological process	210
5	GO:0009058 biosynthesis	51	GO:0007165 signal transduction	209
6	GO:0007165 signal transduction	50	GO:0045045 secretory pathway	170
7	GO:0009628 response to abiotic stimulus	41	GO:0008152 metabolism	166
8	GO:0009607 response to biotic stimulus	36	GO:0009628 response to abiotic stimulus	161
9	GO:0045045 secretory pathway	36	GO:0009058 biosynthesis	155
10	GO:0008152 metabolism	35	GO:0006464 protein modification	148

Supplementary Table S6. Primer sequences used in the study.

Gene Bank accession	Primer Name	Sequence
BJ237316	Lea-F	CTGTTCGCCGTTGCTTTGAT
	Lea-R	CTGGTCCACATTTGAATACTCACG
CK218048	PHO-F	GACGCCAACGGCAGGAAG
	PHO-R	CGAGGGTGACGGCCCAGA
CA613289	Per-F	AACCTGATGTCCCAGAAGGG
	Per-R	GGTGAGCGGGCTGATGTTC
BQ172325	H3-F	GGGCCACTAGCAGTTCAGGG
	H3-R	GGAAGGTGGACTGTCGCTGT
CK214164	Mease-F	GCTCCTCGAACTCCCTCTGG
	Mease-R	CACGACCCTACTAAGGAACTGCTA
AB181991.1	ACT-F	CCTCATGCTATCCTTCGTTTGG
	ACT-R	CGCTCAGCGGTTGTTGTGA
AF139915	WRAB-F	ACACCACCACCAGGAATCA
	WRAB-R	CTGTAGAAGGCTCGTGAACG
U80037.1	PM-19-F	GAAGAAAGAAGCAAGGCAGC
	PM-19-R	TGAAGTGGTTGAGGTTCCAG
BQ295477	H2B-F	AAGTCCAAGAAGAGCGTGG
	H2B-R	GAAGATGTCGTTGATGAAGGAG
